# A Pilot Study on the Effect of Added Sugar on Response Inhibition: Event-Related Potentials in a Go/NoGo Task

**DOI:** 10.3390/medicina61020323

**Published:** 2025-02-12

**Authors:** Karolina Jocbalyte, Rytis Stanikunas

**Affiliations:** Institute of Psychology, Vilnius University, Universiteto 9, LT-01513 Vilnius, Lithuania

**Keywords:** added sugar, cognitive functions, response inhibition, event-related potentials

## Abstract

*Background and Objectives*: Added sugar usage has become an important public health issue nowadays. Therefore, the interest in studying the cognitive and emotional effects associated with sugar consumption has increased. The present study aimed to investigate how the intake of added sugar affects participants’ impulsivity and cognitive functions monitored during the performance of a computerized Go/NoGo task. *Materials and Methods*: This study included 20 subjects (10 men and 10 women). Quantitative data for this study were collected via self-report questionnaires, including demographics, the Dietary Fat and Free Sugar—Short Questionnaire (DFS), the Yale Food Addiction Scale (YFAS 2.0), and the Barratt impulsiveness scale-11 (BIS-11). *Results*: Event-related potentials (ERPs) were registered throughout this study. Comparing the results of psychophysiological and neuropsychological evaluations before and after the added sugar consumption reveals differences in ERPs. Specifically, the NoGo-P3 amplitude increased after the intake of added sugar. However, there were no behavioral differences between the two experimental sessions. *Conclusions*: Overall, the results of our study suggest that added sugar intake was associated with stronger neuronal firing in NoGo trials. One possible explanation for this could be the need for more cognitive endeavors for participants to successfully inhibit their response impulses after added sugar consumption.

## 1. Introduction

According to the World Health Organization (WHO) statistics, despite the recent pandemic situation, the main cause of death continues to be non-communicable diseases (ischemic heart disease, stroke and heart attack, cancer, diabetes, etc.). Unhealthy lifestyles and dietary habits are important factors contributing to all these conditions. Poor dietary habits and malnutrition are usually associated with eating highly processed foods that are rich in fat and added sugar. Added sugars or free sugars refer to all monosaccharides and disaccharides that are added to foods by the manufacturer or by the consumer. It includes several chemical forms: sucrose, glucose, fructose, dextrose, etc. [1]. It is recommended that added or free sugar intake should be less than 10% of total energy intake. Nevertheless, data from several studies in different countries suggest that people rarely follow these guidelines, as the immediate enjoyment of sugar’s taste is often valued more than its potential health risks [2].

People often see sugar as a key energy source essential for proper brain function. However, the results of studies on the acute effects of sugar ingestion on cognitive functions are highly controversial and depend on the assessed cognitive domain and methods that were used. Glucose ingestion has been found to have a facilitating effect on memory tasks, but results about its effects on reaction time, attention, working memory, and related types of tasks are mixed [3]. Likewise, scientific research shows that long-term added sugar consumption is associated with cognitive impairments [4,5,6].

Although some studies showed that participants had little knowledge about sugar intake recommendations, usually, this is not the case. Naturally, the question arises—why, despite the serious consequences for their well-being, do people make unhealthy diet choices? Research into malnutrition, eating disorders, and obesity has identified several factors that encourage sugar consumption [7,8]. As a result, several individual and contextual factors were identified as promoting sugar consumption. The most relevant individual factors were habit, enhanced flavor or pleasure, negative events, and emotions, whereas social influence, limited time to eat, and limited variety of available food were the most referred contextual factors [2].

Moreover, added sugar has other important properties that are closely related to the stress hormone system [9]. The potential of added sugar to alter emotional state can be highly reinforcing. Recent studies indicate that palatable foods high in added sugar have the capacity to rewire brain reward circuits [10]. Multiple studies show that eating high-calorie, sweet foods stimulates the dopaminergic brain system and affects the reward and pleasure centers that encourage repetitive behavior [11,12]. In addition, palatable food, and sugar in particular, acts on endogenous opioids, activating mu-opioid and kappa-opioid receptors [13,14]. That might explain why opioid-dependent people often report greater consumption of unhealthy and sweet foods and increased sugar cravings [15]. These findings reveal the addictive properties of added sugar and contribute to the idea of food addiction.

Research in food addiction is growing, with recent reviews suggesting that food addiction is a distinctive condition that has many symptoms resembling substance use disorders: compulsive use, withdrawal syndrome, and increased sensitivity to other psychoactive substances. The evidence supports the hypothesis that under certain circumstances, rats can become sugar-dependent. This may translate to some human conditions, as suggested by the literature on eating disorders and obesity [16]. To sum up, while there are differences between the addictive characteristics of food and illicit substances, there are many parallels that should not be ignored [17]. Thus, research investigating this area in more detail would be beneficial.

It is known that individuals suffering from addictive disorders show deficits in response selection and inhibition of unwanted automatic responses that are a part of the adaptive cognitive control system. Studies revealed a correlation between low impulse control and addictive disorders. What is more, it includes not only individuals with substance dependence but also those showing addictive behaviors like pathological gambling, gaming, and excessive eating [18,19]. The most common way to assess the ability to stop or withhold inappropriate responses in a laboratory environment is through a Go/NoGo task in which participants must respond quickly to frequently occurring Go stimuli and inhibit responses while presented with rare NoGo stimuli. Reduced NoGo accuracy was shown in individuals with behavioral addictions [20].

Response inhibition in the Go/NoGo task is consistently linked with two major components of ERPs: NoGo N2 and NoGo P3. The negative component (NoGo-N2) is related to the process of conflict monitoring. Meanwhile, the positive wave (NoGo P3) is related to response inhibition and indexes a late stage of the inhibitory process, such as response evaluation or successful response inhibition [18]. It is believed that a significant variation in these components is related to some degree of inhibitory deficiency.

However, the results of studies examining ERPs in behavioral and substance addictions are contradictory. A review of such studies found that the results of studies on P3 amplitudes are inconsistent. Some studies showed no differences between individuals with addictions and controls, whereas other studies showed lower or higher P3 amplitudes in those with addictions [19]. Therefore, more research in this area would be crucial.

The current pilot study explored the influence of added sugar on healthy subjects’ response inhibition while performing the Go/NoGo task. A within-participants comparison was conducted by comparing the results of the same subjects before and after sugar consumption. The aim of our investigation was to assess whether and how sugar consumption was related to impulse control based on behavioral and electrophysiological evidence. We hypothesize that added sugar consumption influences impulse control and response inhibition, as measured by behavioral performance and electrophysiological evaluations (ERPs) during a Go/NoGo task. This pilot study would provide preliminary data to refine our hypothesis, assess the feasibility of the experimental design, identify any methodological challenges, and determine the appropriate sample size for future research.

## 2. Materials and Methods

### 2.1. Participants

A total of 20 subjects (10 males and 10 females) participated in this study. They ranged in age from 20 to 38 years (*M* = 26.50, *SD* = 6.16). Based on self-reports, all subjects had no apparent health problems, were free from previous head injuries, and were not using any substances that might affect their physiological or psychological state. The average body mass index (BMI) of the participants was 23.84 (*SD* = 4.05). All participants provided written informed consent for participation in the current study. The protocol of this study was approved by the research ethics committee of Vilnius University.

### 2.2. Measurements

Participants provided general information by completing the demographic survey. They also filled in the Dietary Fat and Sugar Questionnaire (DFS) [21]—a brief food frequency questionnaire (scores ranging from 26 to 130) that measures added sugar and saturated fat intake. It captures foods that are not consumed every day but are part of a typical person’s diet. In the current sample, the DFS demonstrated a good total internal reliability because of a Cronbach α of 0.894.

Food addiction-related symptomology, such as decreased consumption control, persistent cravings, or repeated unsuccessful attempts to discontinue the behavior, was assessed using the Yale Food Addiction Scale 2.0 (YFAS 2.0) [22]. Subjects had to have at least one clinically significant symptom to be classified as food addicted. The severity of the food addiction was defined as mild (2 or 3 symptoms), moderate (4 or 5 symptoms), or severe (6 or more symptoms). If participants self-reported none or only one symptom, they would be classified as not having food addiction. In the current sample, the YFAS 2.0 demonstrated a good total internal reliability because of a Cronbach α of 0.890.

The Barratt Impulsiveness Scale-11 (BIS-11) [23] was applied to assess impulsivity. It is a self-assessment questionnaire frequently used in clinical and research studies. The scale consists of 30 questions describing impulsive or non-impulsive (inversely assessed elements) behaviors and desires. The total score resulting from the sum of the second-order subscales of the BIS-11 was derived. The BIS-11 demonstrated a relatively low internal reliability in the current sample because of a Cronbach α of 0.675.

Finally, in our study, we used the Go/NoGo task. It was similar to the one that was used in the Kilian et al. (2020) study [20]. A white circle was presented on a gray background throughout the task. After a variable inter-stimulus interval of 900 to 1200 ms in the center of the circle, either a green or a red square was displayed for 600 ms. The green square appeared in 75% of all trials and was a Go stimulus. Participants had to respond to it as quickly as possible by pressing the button “1” on a keyboard. The red square was shown in 25% of all trials. It was a NoGo stimulus, and participants had to withhold their response. In our study, the Go/NoGo task consisted of 100 trials. Completion of the task took about 5 min. EEG was recorded during the entire task trial, and time-locked EEG activity or ERPs were calculated.

During the task in both sessions, EEG was recorded using a BIOPAC MP150 System. AcqKnowledge 4.4 software was used for recording and real-time monitoring of EEG data. Four recording channels were used. The EEG was recorded at 4 scalp sites (FPz, Cz, Pz, and O2) positioned according to the international 10–20 system using silver/silver chloride (Ag/AgCl) electrodes. These sites offer a view of frontal, central, parietal, and occipital regions, allowing the capture of cognitive processes relevant to the pilot study while maintaining a manageable number of channels. Reference electrodes were placed on earlobes. EEG signals were obtained with a band-pass filter of 0.1–100 Hz. The EEG was sampled continuously at a rate of 2000 Hz. During the recording, impedances were kept below 5 kΩ.

### 2.3. Procedure

**Figure 1:**
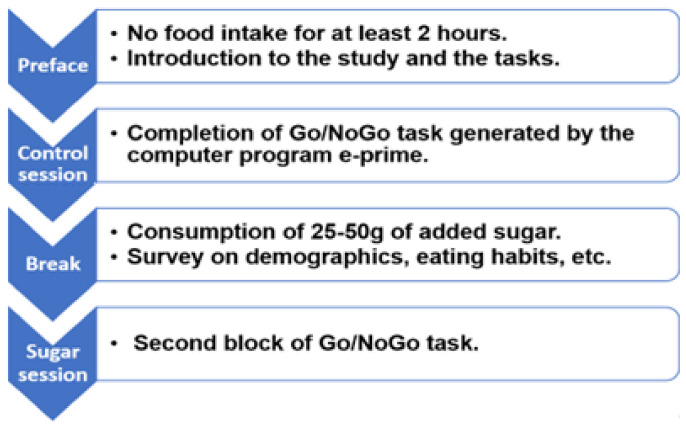
Experimental procedure.

Participants were instructed to abstain from any food or drinks (except water) for at least two hours before the session. Then, they were introduced to the study’s design and procedures and completed a survey on demographics. To avoid an order effect and ensure that participants are equally well trained in both sessions, they received pre-experiment training. During the training phase, participants completed a learning task. This training task was untimed, provided continuous feedback on performance, and closely mirrored the experimental task to ensure effective skill acquisition. On average, participants completed between 10 and 15 training trials, although the exact number varied depending on individual performance. Participant self-assurance was considered in tandem with objective performance assessment. During the task, subjects were seated in a quiet room facing a computer monitor placed at a distance of 60 cm from the participant’s face. First, the control session was completed. During it, participants underwent the first block of Go/NoGo trials generated by the computer program e-prime. Then, the participants consumed an added sugar. They were asked to drink a sweet beverage containing 25–50 g (depending on their body mass, approximately 1 g of sugar for 2 kg of the body mass) of normal table sugar and rested for 15–20 min to allow enough time for the sugar to take its effect. During that period, questionnaires were filled in. Finally, they performed a second block of Go/NoGo tasks. Both blocks of the Go/NoGo tasks consisted of 100 trials. Throughout both sessions, EEG was recorded with the BIOPAC MP150 System. The scheme of the procedure is shown in the figure below (Figure 1). The results were compared for each subject before and after the consumption of an added sugar.

### 2.4. Data Analysis

Data were preprocessed and analyzed using EEGLAB in MATLAB [24] with the additional plugin Darbeliai [25]. During preprocessing of the data, the sampling rate was reduced to 256 Hz (to optimize the calculation process). The data were then band-pass-filtered between 0.3 and 40 Hz to suppress electrical line noise. Segments containing artifacts were removed by visual inspection. Only results low on artifacts were used for averaging. In total, fewer than 5% of the data were excluded.

For ERP analysis, epochs spanning from 200 ms before to 1000 ms after the stimulus onset were obtained. The baseline was defined as EEG activity during the 300 ms interval preceding the stimulus onset, and it was subtracted from the data. The amplitudes for the electrode locations FPz, Cz, Pz, and O2 were calculated. After averaging, evoked potentials were visually inspected, and time windows were selected for further analysis. The most negative (or the smallest) value in the time window from 170 to 220 ms after stimulus onset was defined as N2. The positive wave appearing from 300 to 450 ms after stimulus onset was defined as P3.

Further statistical analysis was performed using the IBM SPSS Statistics 22.0 software. Student’s *t*-test for paired samples was conducted for the same dependent variables of each subject (reaction time, number of errors, and main ERP effects for the four channels). These variables measured during the first session (before the added sugar consumption) and the sugar session (after the added sugar consumption) were compared. To test the relationship between results from the questionnaires (demographic, BIS-11, YFAS 2.0, DFS), behavioral results (reaction time, number of errors), and main ERP effects that occurred after sugar consumption, regression analysis was conducted.

To improve grammar and clarity, AI-based grammar-checking and language-enhancement tools (such as ChatGPT and Grammarly) were used in the manuscript preparation process. However, all intellectual contributions and interpretations remain the responsibility of the authors.

## 3. Results

It is known that averaging the signals from multiple electrodes in ERP analysis can help improve the signal-to-noise ratio, particularly when focusing on components that are recorded from several electrodes, such as P3 and N2. Measuring from a multi-site cluster of electrodes yielded results as good as or better than a single electrode, suggesting that cluster-based measurement is effective for quantifying ERP [26]. For this reason, we used an average of four electrodes for our analysis. In Table 1, the amplitudes of ERP components and their latencies during the two experimental sessions are shown. There were no differences in the N2 components of ERPs. The amplitude and latency were not statistically significantly different between the two sessions. In numerous studies, an enhanced N2 amplitude has been associated with conflict detection. Thus, this suggests that subjects were equally effective at detecting conflict in both sessions.

**Figure 2:**
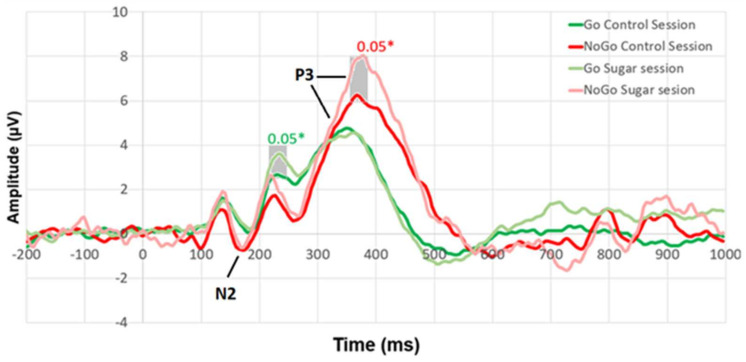
The ERP average calculated from all four channels recorded during the Go/NoGo task. * Statistically significant differences, *p* < 0.05.

On the other hand, there were differences between the NoGo P3 components. These differences were observed both by calculating the absolute peaks (Table 1) and by comparing the curves of ERPs at each time point (Figure 2).

**Figure 3:**
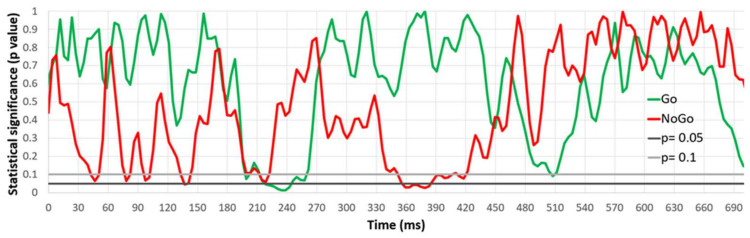
Level of significance of difference between the ERP averages for all four channels recorded during the control and sugar sessions.

Figure 2 shows the participant responses to different stimuli (reaction to Go stimuli (dark green line), NoGo stimuli (red line) during the control session, and Go stimuli (light green line), NoGo stimuli (light red line) during the sugar session). Student’s *t*-test for paired samples was conducted for the control and sugar session ERPs at each time point. Statistically significant differences between the ERPs and their significance levels are marked on the graph. Figure 3 shows that there were statistically significant differences (*p* < 0.05) in P3 (355–385 ms) while reacting to NoGo stimuli. In addition, a statistically significant difference was found in ERP around 215–245 ms when reacting to Go stimuli.

**Figure 4:**
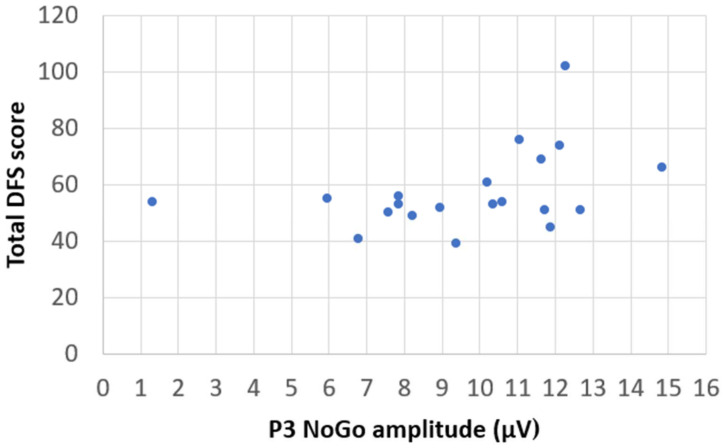
The relationship between the DFS estimates and NoGo P3 amplitude in sugar session.

We aimed to examine how ERP amplitudes (eight variables: Go-N2, NoGo-N2, Go-P3, NoGo P3 in control session and Go N2, NoGo N2, Go P3, NoGo P3 in sugar session) were related to various participant characteristics assessed by different questionnaires (nine variables were considered: total YFAS score, attentional, motor, non-planning, and total impulsiveness, as measured by BIS-11, as well as fat, sugar, fatty-sugar, and total score, as measured by DFS). The pilot study revealed a statistically significant relationship (correlation r = 0.391, *p* = 0.044) between the DFS estimates and NoGo P3 amplitude in the sugar session. A trend was observed, suggesting that the higher the NoGo P3 amplitude during the sugar session, the more sugar the subject consumed according to their DFS score (Figure 4).

**Figure 5:**
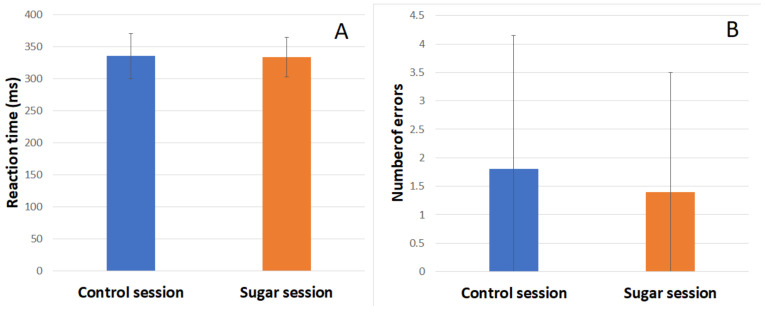
Behavioral results of the Go/NoGo task in the control and sugar sessions: (**A**) Average reaction time across two sessions and its standard deviation (*SD*). (**B**) Average number of errors when subjects impulsively pressed the button in NoGo situations across the control and sugar sessions and its standard deviation (*SD*).

During both sessions, subjects’ reaction time (RT) and number of errors were recorded. Behavioral results of the Go/NoGo task in the control session and sugar session are shown in the figure below (Figure 5). There was no significant difference in the RT or number of errors between the two sessions.

A separate comparison of how these results were related to other participants’ characteristics recorded during the study revealed that RT in both sessions was related to age. The older the person was, the longer the RT was. The results of the regression indicate that age explained 41% of the variation in RT in the control session and 38% in the sugar session [*F*(1,18) = 12.573 (norm) and 10.969 (sugar), *p* < 0.01].

RT in the sugar session was also related to BIS-11-measured attentional impulsivity and total impulsivity. The higher the attentional impulsivity or total impulsivity score, the longer the RT after added sugar consumption. The results of the regression indicate that attentional impulsivity explained 24.4% of the variation in RT in sugar sessions [*F*(1,18) = 5.800, *p* = 0.027]. The same relationship is observed with the overall impulsivity score. The results of the regression indicate that total impulsivity explained 20.8% of the variation in RT in sugar sessions [*F*(1,18) = 4.723, *p* = 0.043].

In our pilot study, RT in the sugar session could be determined by the overall estimate of YFAS 2.0. The subjects who subjectively noticed more food addiction-related symptoms in themselves and received higher YFAS 2.0 scores responded more slowly to stimuli after consuming the added sugar. Results of the regression indicate that the YFAS 2.0 score explained 18.9% of the variation in RT in sugar sessions [*F*(1,18) = 4.205, *p* = 0.055].

## 4. Discussion

It is often assumed that the added sugar has a positive effect on cognitive functions immediately after its consumption [27]. If so, it would be seen in behavioral performance, like smaller RT and fewer errors in the same cognitive tasks after the added sugar consumption. However, we did not obtain such results in our study. Comparing the behavioral results of the Go/NoGo task before and after the added sugar consumption revealed no differences in reaction time or number of errors between the two sessions.

On the other hand, differences in psychophysiological reactions were observed, and the ERPs revealed significant differences in the P3 components. Usually, less pronounced N2 or P3 amplitudes are observed in addicted populations compared with controls [19]. However, these reduced amplitudes are also associated with poorer behavioral performance, which allows them to be considered markers of neural deficits in inhibitory control.

In our case, there were no behavioral differences between the two conditions (the participants’ RT and the number of errors were the same), but there were differences in ERPs—the higher amplitude of NoGo P3 after the added sugar consumption. It is thought that NoGo P3 is an index of response inhibition. Hyperactivation coupled with intact behavioral performance is often interpreted as increased neural effort or the use of alternative cognitive strategies to achieve normal levels of behavioral performance [19]. As no behavioral deficits were found in the sugar session, this may reflect the need for more cognitive efforts for successful inhibition of response impulses or, rather, more effort to refrain from making an incorrect response when the red square appeared.

Our results are compatible with Dong et al. (2010), who monitored impulse inhibition while registering ERPs during the Go/NoGo task in people with Internet addiction results. In their study, less pronounced N2 and larger P3 components were observed among excessive Internet users. The authors explained that enhanced activation in the final stage of inhibitory control could have served as compensation for the less efficient early inhibitory mechanisms in excessive Internet users to obtain behavioral performance levels equal to those of casual Internet users [18]. However, we did not observe any difference in the N2 component.

Stronger neural excitation (not related to better task performance) has also been observed in participants with eating disorders. In a study by Lock and colleagues, where they used fMRI and measured brain activation during the Go/NoGo task in eating disorder subtypes and control groups, similar behavioral accuracy levels were found. On the other hand, participants with binge eating behavior had more brain activation associated with inhibitory control (such as the right DLPFC, right ACC, bilateral precentral gyri, bilateral hypothalamus, and right MTG) compared with the control group [28].

Larger P3 waves can also be interpreted the other way around, that in the first part of the study, the subjects saved energy. Study findings revealed that the metabolic state dynamically regulates the energy spent on coding precision [29]. Therefore, it would be worthwhile to compare results between two sessions with a third session, where subjects would be required to fast for a longer period of time.

Recent studies suggest that food addiction has many symptoms similar to substance use disorders, and interest in it is increasing [30]. The current study suggests that in the post-sugar session, participants may have required greater cognitive effort to inhibit impulsive responses with some variation based on usual sugar intake levels (as indicated by DFS estimates). We observed that NoGo P3 amplitude appeared to increase in the sugar session, showing an association with a larger sugar intake indicated by DFS. The instantaneous effect of added sugar seemed to vary according to the everyday quantity of added sugar in the participants’ diet. Subjects who used to consume more added sugar did not differ according to their RT or number of errors. On the other hand, added sugar consumption showed a possible relationship to larger NoGo P3 amplitudes. These findings could suggest that they were more impulsive and needed more effort to refrain from responding. However, the subjects in our study were quite homogeneous, and their DFS estimates were close to average. Future interventions could consider a more diverse sample of subjects with very low or very high daily added sugar intakes (according to DFS results).

Moreover, there were several additional variables that we did not control in this study. For example, subjects’ attitudes towards added sugar and beliefs of how their results should be affected by the consumption of this substance. It is known that the effects of food, and added sugar in particular, can be affected by an individual’s beliefs about it [31]. Finally, the small sample size should also be taken into consideration while interpreting the results. Further research could extend these findings.

## 5. Conclusions

It is commonly believed that added sugar has an immediate positive effect on cognitive performance. What is more, glucose is indeed an essential energy source for the brain and can support cognitive functions, for example, have a facilitating effect on memory tasks [3]. However, our pilot study prompts us to check this assumption more carefully. Subjects who participated in the experiment showed no significant differences in RT and error rates before and after sugar consumption. However, differences were observed in psychophysiological responses, specifically in the P3 component in NoGo trials. A larger NoGo P3 amplitude after sugar consumption without accompanying behavioral deficits may suggest that participants had required greater cognitive effort to maintain their performance levels. The results align with previous studies on behavioral addictions, where similar patterns of heightened neural effort were observed.

This pilot study’s findings hint at a possible relationship between habitual sugar intake and NoGo P3 amplitudes, as participants with higher regular sugar consumption appeared to show larger amplitudes. However, this association was not statistically significant and would require further investigation to clarify. This could indicate a greater impulsivity or the need for more effort to inhibit inappropriate responses. Future research should explore the effect of added sugar in a more diverse sample (particularly those with extreme sugar consumption). This is important, as this pilot study suggested the need to account for variations in sugar consumption and related factors. Moreover, this pilot study helped to identify other factors that may influence the effects of sugar, such as the role of participants’ attitudes and beliefs about sugar intake. Further investigations could provide valuable insights into the complex relationship between sugar intake, cognitive performance, and neural activity.

## Figures and Tables

**Table 1:** The absolute N2 and P3 peaks and their latencies across two sessions.

		N2 Amplitude (µV)	P3 Amplitude (µV)	N2 Latency (ms)	P3 Latency (ms)
Control session Mean (± SD)	Go stimulus	−0.61 (±0.002)	6.08 (±0.003)	188.086 (±14.795)	343.945 (±31.390)
	NoGo stimulus	−1.99 (±0.003)	7.85 * (±0.004)	183.008 (±18.681)	364.944 (±40.504)
Sugar session Mean (± SD)	Go stimulus	−0.59 (±0.003)	6.16 (±0.002)	185.547 (±13.738)	341.992 (±31.797)
	NoGo stimulus	−1.42 (±0.003)	9.66 * (±0.003)	184.375 (±18.304)	366.211 (±36.575)
Statistical significance	Go stimulus	0.956	0.892	0.217	0.788
	NoGo stimulus	0.246	0.033 *	0.707	0.870

* Statistically significant differences, *p* < 0.05.

## Data Availability

The dataset obtained in the current study is available from the authors upon request.
